# Keep your finger on the pulse: Better rate perception and gap detection with vibrotactile compared to visual stimuli

**DOI:** 10.3758/s13414-023-02736-y

**Published:** 2023-08-16

**Authors:** Mercedes B. Villalonga, Robert Sekuler

**Affiliations:** 1 Department of Psychology, Brandeis University, Waltham, MA USA; 2 Program in Neuroscience, Brandeis University, Waltham, MA USA

**Keywords:** Vibrotactile, Temporal perception, Temporal variability, Interval timing, Rate perception, Multisensory integration.

## Abstract

Important characteristics of the environment can be represented in the temporal pattern of sensory stimulation. In two experiments, we compared accuracy of temporal processing by different modalities. Experiment 1 examined binary categorization of rate for visual (**V**) or vibrotactile (**T**) stimulus pulses presented at either 4 or 6 Hz. Inter-pulse intervals were either constant or variable, perturbed by random Gaussian variates. Subjects categorized the rate of **T** pulse sequences more accurately than **V** sequences. In **V** conditions only, subjects disproportionately tended to mis-categorize 4-Hz pulse rates, for all but the most variable sequences. In Experiment 2, we compared gap detection thresholds across modalities, using the same **V** and **T** pulses from Experiment 1, as well as with bimodal (**VT**) pulses. Visual gap detection thresholds were larger (3$\times$) than tactile thresholds. Additionally, performance with **VT** stimuli seemed to be nearly completely dominated by their **T** components. Together, these results suggest (i) that vibrotactile temporal acuity surpasses visual temporal acuity, and (ii) that vibrotactile stimulation has considerable, untapped potential to convey temporal information like that needed for eyes-free alerting signals.

**Supplementary Information:**

The online version contains supplementary material available at 10.3758/s13414-023-02736-y.

## Introduction

Important characteristics of the environment can be represented in temporal patterns of stimulation within multiple sensory modalities. Much is known about the eyes’ and ears’ ability to process temporal stimulation, but relatively little is known about the processing capacity of our largest sensory organ, the skin. Despite that limited knowledge, various applications make use of time-varying vibrotactile cues. For example, a mobile phone’s distinctive pattern of vibrations can identify the caller; vibrations from a smart watch can remind the wearer to perform some action; and vibrations from a car’s steering wheel can warn of a possible collision (Chiasson et al., 2003; Elliott et al., 2009; McGrath et al., 2004). These and other potential uses of the skin as a communication channel make it important to know the skin’s capacity to process temporal patterns of stimulation, and how limitations on that capacity compare to other sensory channels.

It is well established that auditory temporal processing surpasses both tactile and visual temporal processing (Desloge et al., 2014; Jones et al., 2009; Rammsayer, 2014; Recanzone, 2003; Stauffer et al., 2012; Villalonga et al., 2021; Welch et al., 1986). Fewer psychophysical studies have directly compared tactile and visual temporal processing, and these visuo-tactile comparisons yield mixed results. Subjects tend to have slightly higher sensitivity to temporal information conveyed by tactile signals compared to visual (Azari et al., 2020; Ball et al., 2017; Bresciani et al., 2008), although there is also evidence of higher temporal sensitivity with visual signals than with tactile signals (Grondin & Rousseau, 1991). Notably, differences between visual and tactile temporal sensitivity generally either fail to reach statistical significance (Ball et al., 2017; Jones et al., 2009), or are easily negated by changing the task parameters (*e.g.*, unpredictably varying stimulus modality from trial to trial, or using time intervals >1 s; see Azari et al. , 2020; Grondin & Rousseau, 1991).

Many previous studies of temporal sensitivity asked subjects to judge the duration of single stimuli. It is not guaranteed that results with judgments of single intervals can be generalized to more complex temporal stimuli such as temporal frequency or rate (Breska & Ivry, 2018; Rammsayer & Brandler, 2004; Teki et al., 2011). Temporal frequency is the rhythmic pattern of stimulation typically described as a beat-based temporal structure. A recent study suggested that perceived rate is built up over time from the perceived durations of component intervals (Motala et al., 2020). This finding suggests interval-based timing mechanisms contribute to rate perception, and that modality differences in interval timing could also affect perception of more complex temporal structures.

In a recent study, we investigated how stimulus modality affected ability to classify pulse rates as “fast” (6 Hz) or “slow” (3 Hz), as well as the impact of each inter-pulse interval on rate perception (Villalonga et al., 2020). With stimuli delivered as trains of either vibrotactile or visual pulses, subjects categorized stimulus rate equally well with either modality of stimulation. That result held even with non-isochronous pulse trains (*i.e.*, when the regularity in timing of successive pulses was degraded by additive temporal noise). Interestingly, the earliest inter-pulse intervals in each pulse sequence had a larger impact on accurate rate categorization than later intervals.

In the present study, we tested whether more challenging conditions would produce similar results, and whether sensitivity to individual intervals between pulses plays a role in rate perception. Specifically, we tested temporal acuity for unfilled intervals across visual and tactile modalities, taking convergent approaches in a pair of experiments. Experiment 1, a rate categorization task, showed that sensitivity to rate information depends upon sensory modality, with improved performance on tactile trials compared to visual. Experiment 2 extended this finding to gap detection: visual gap detection thresholds were significantly larger than tactile thresholds, and bimodal thresholds were largely dominated by tactile components.

## Experiment 1: Using Temporal Noise to Investigate Rate Processing

Several different sources of noise can corrupt sensory responses. Although intrinsic noise is present in any perceptual process, external noise added to a stimulus under controlled conditions can reveal the computations governing perception of that stimulus (Allard et al., 2015; Pelli & Farell, 1999). To quantify perceptual efficiency (Lu & Dosher, 1999) or to characterize information processing during particular tasks (Gold, 2014; Gold et al., 2004), vision researchers have injected noise into luminance (Gold, 2014; Hall et al., 2014) and orientation stimuli (Girshick et al., 2011). We adapted this approach to compare temporal processing of tactile and visual stimuli. Specifically, we injected varying amounts of temporal noise into both visual and vibrotactile stimulus sequences. Subjects were required to categorize each sequence as having a mean rate of either 4 or 6 Hz. On some trials, the sequence of pulses was isochronous (*i.e.*, temporally regular), but on most trials, this temporal regularity was perturbed by random variation in the timing of pulses. We asked whether increasing amounts of temporal noise would differentially impair rate categorization in the two modalities, revealing a differential robustness in the modalities’ capacity to transmit rate information.

### Method

#### Subjects

Twenty-eight subjects were tested (20 female, 6 male, 2 declined to identify; mean age = 18.7 years, SD = 0.9). This sample size gave us 88.3% power to observe an effect size comparable to that in our previous, related experiment ($\eta _p^2$ = 0.16; Villalonga et al. , 2020). Experimental procedures were approved by Brandeis University’s Institutional Review Board and were conducted in accordance with the Declaration of Helsinki. Subjects gave written informed consent prior to participation.

#### Apparatus and Stimuli

The flow of the experiment and stimulus delivery was controlled by PsychoPy software (Peirce et al., 2019) running on an Apple Mac Mini computer. The delivery and timing of stimulus pulses were controlled by an Arduino microcontroller (Banzi & Shiloh, 2014) that communicated with the computer via serial port.

**Fig. 1:**
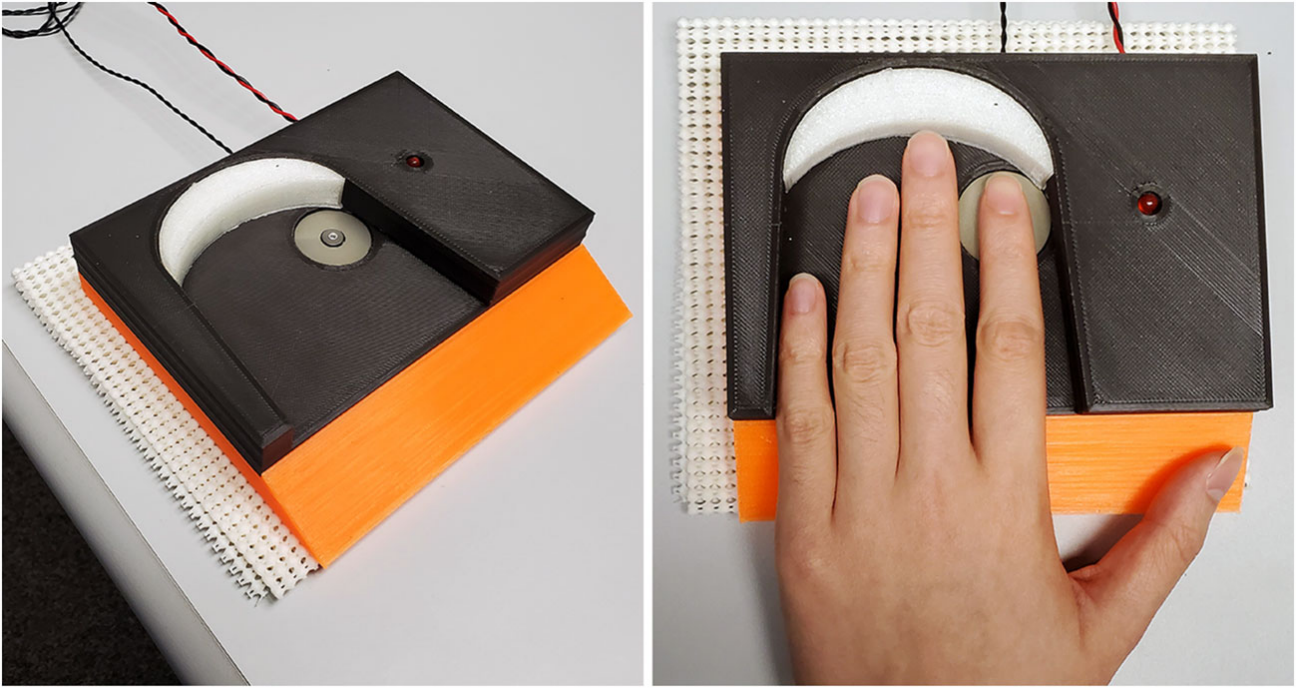
Apparatus for stimulus presentation. The LED and tactor were embedded in a bespoke hand cradle and used for presentation of visual and vibrotactile stimuli, respectively. Multiple interchangeable components in the hand cradle allowed us to accommodate hands of different widths and finger lengths in order to ensure that the tip of a subject’s index finger was positioned atop the vibrating tactor.

**Fig. 2:**
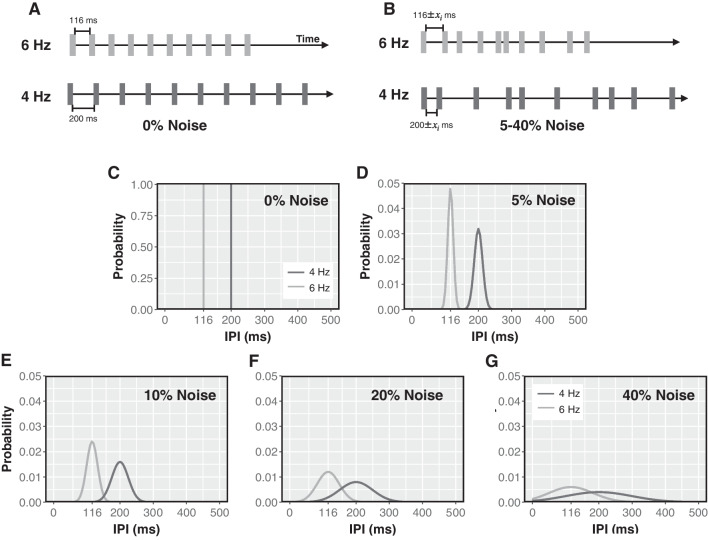
In Experiment 1, series of 50-ms pulses (grey vertical bars, **A** & **B**) were presented on each trial. The stimulus terminated when either 10 pulses had been presented or if the subject made a response. Pulse sequences presented in the 0% noise condition (**A**) were isochronous, with each IPI fixed at a nominal IPI for the given rate (**C**). On trials with noise, each nominal IPI in the trial sequence was independently perturbed by a random variate (**B**). Scaling noise by the nominal rate produced IPI sampling distributions for 4-Hz and 6-Hz trials that were unequal in variance (**D**-**G**). Sampling distributions were truncated to prevent IPIs $\le 1$ ms. Note: Diagrams of pulse sequences (**A** & **B**) are not drawn to scale.

Vibrotactile (**T**) pulses, each 50 ms long, were delivered to the distal pad of the subject’s left index finger using a C-2 tactor (Engineering Acoustics, Inc., Casselberry, FL). Pulses were generated by driving a 7.87-mm diameter, metal tactor element up and down against the skin at 250 Hz. The Arduino controlled activation of the tactor via a DRV2605L Haptic Motor Driver board (Texas Instruments, Dallas, TX, USA). Visual (**V**) pulses were 50-ms flashes produced by a 5-mm diameter LED with a red lens. The initial, positive phase of vision’s temporal impulse response function grows more compressed in time with increasing mean luminance (Kelly, 1971). So, to optimize the precision of visual responses to our stimulus pulses, LED luminance was set at a value in the upper end of its safe operating range. As measured by filling the 1 degree field of a Minolta LS-1000 photometer, mean LED luminance was 968.5 cd/m$^2$.

The tactor and LED were both fixed in the adjustable, 3D-printed hand cradle, shown in Fig. 1. The LED’s spatial adjacency to the tactor promoted approximate co-localization of visual and vibrotactile stimuli (Badde et al., 2018). The cradle held a subject’s left hand comfortably, ensuring that the tip of the index finger was located directly on the tactor. Removable 3D-printed inserts adjusted the hand cradle’s dimensions for best fit to individual subjects’ hands (Donelson & Gordon, 1995).

Subjects were monitored throughout the experiment to ensure that they kept their finger in the correct position on the tactor, with their eyes open. Subjects were instructed to fixate on the LED throughout the experiment. To prevent auditory signals generated by the tactor’s vibrations from impacting subjects’ responses, masking white noise was delivered to subjects over noise-canceling headphones.

On each trial, subjects received a sequence of 50-ms stimulus pulses separated by empty inter-pulse intervals (IPIs; Fig. 2). All pulses on a single trial were presented either visually, as LED flashes, or as vibrotactile pulses to the finger tip. Equal numbers of sequences with the two mean rates, 4 or 6 Hz, were randomly intermixed across trials. On 20% of trials, pulses were isochronous: each 50-ms pulse was separated from the next by either 200-ms IPIs (4-Hz trials) or 116-ms IPIs (6-Hz trials; Fig. 2A). On the other 80% of trials, pulse sequences were temporally stochastic, with a random variate (hereafter, "noise") added to each IPI in a sequence (Fig. 2B). The noise $x_i$ was generated by sampling zero-mean Gaussian distributions, $x_i \sim N(0,\sigma ^{2})$, where $\sigma$ varied by both noise level and pulse rate. For the isochronous, 0% noise condition, $\sigma$ = 0 (Fig. 2C). Non-isochronous sequences were chosen randomly from stochastic sequences with one of four noise levels: 5, 10, 20, and 40% noise. These four noise levels were operationally defined by scaling the variance of the IPI sampling distribution, where $\sigma$ = 0.05*k*, 0.1*k*, 0.2*k*, and 0.4*k*, respectively, with *k* being the nominal period in milliseconds for the rate condition (4 Hz: *k* = 250; 6 Hz: *k* = 166). Variates were sampled independently for each IPI in a sequence and for each trial. The sampled distributions were truncated at the lower tail to prevent IPIs $\le$1 ms. Figure 2 depicts the IPI sampling distributions for each noise level (panels C-G).

#### Task

A 500-ms tone (440 Hz) alerted the subject to the start of a trial. Then, 500 ms later, the trial’s pulse sequence began and continued until the subject responded or until 10 pulses (nine IPIs) had been presented. The maximum number of pulses provided on each trial was determined based on previous work in our laboratory that showed subjects invariably needed fewer than 10 pulses to categorize a sequence’s rate (Villalonga et al., 2020). Subjects responded by categorizing the pulse rate as “slow” or “fast", which were the correct responses for 4-Hz and 6-Hz trials, respectively.

Subjects communicated their judgments by pressing one of two computer keyboard keys with their free, right hand. The keys assigned to “slow” and “fast” judgments were counterbalanced between subjects. Subjects were instructed to respond as soon as they made a decision about the pulse rate, and could therefore end the trial before the entire stimulus (10 pulses separated by 9 IPIs) had been presented. Including the time elapsed by the stimulus itself (on average, 1.80 s for 4-Hz stimuli and 1.54 s for 6-Hz stimuli), subjects were allowed up to 8 s to respond, after which the trial automatically ended. Successive trials were separated by an inter-trial interval of 1 s.

To learn the response mapping, each subject first completed a practice block of 20 trials with feedback after each trial. Pulse modality and rate were randomized across trials of the practice block, but noise was held constant at 0% to familiarize subjects with the stimuli and task. Following the practice block, subjects completed 16 test blocks of 50 trials each, yielding a total of 800 test trials from each subject. Trials were blocked by modality, alternating between **V** and **T** from block to block. Block order was counterbalanced between subjects. Pulse rate and noise level were randomized from trial to trial within each block.

#### Data Analysis

Data were analyzed in R (R Core Team, 2021). Trials on which a subject failed to respond were excluded from analysis (*n*=95, 0.4% of trials). Trials with premature responses, defined as a response that occurred before the onset of the second pulse in the sequence (*i.e.*, a response without any rate information), were also excluded (*n*=47, 0.2% of trials).

**Fig. 3:**
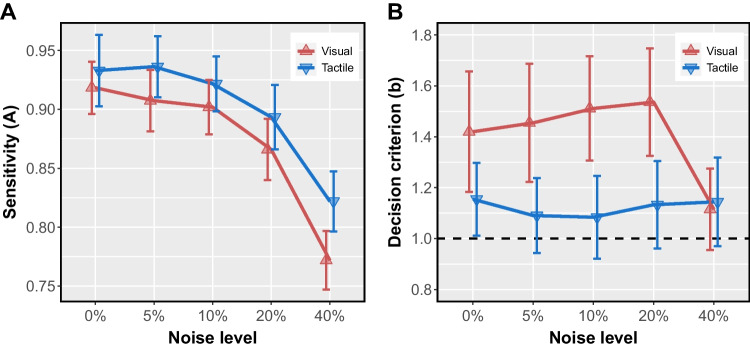
Sensitivity (**A**) and decision criterion (**B**) by noise level for each modality. On average, subjects were better able to categorize rate information conveyed by **T** pulse sequences than by **V** pulse sequences. Subjects’ decision criteria varied between modalities, revealing they were more likely to erroneously categorize **V** sequences as “fast”. Error bars reflect 95% confidence intervals, n=28.

**Table 1:** Two-way (modality $\times$ noise level), within-subjects ANOVAs on sensitivity (*A*) and decision criterion (*b*).

	Sensitivity (*A*)				Decision criterion (*b*)			
Effect	df	F	p	$\eta ^2_p$	df	F	p	$\eta ^2_p$
Modality	1, 27	9.98	**0.004 **	0.27	1, 27	13.62	**0.001 **	0.34
0% Noise	1, 27	1.65	0.210	0.06	1, 27	10.36	**0.003**	0.28
5% Noise	1, 27	10.25	**0.004**	0.28	1, 27	14.47	$\mathbf {< 0.001}$	0.35
10% Noise	1, 27	2.74	0.109	0.09	1, 27	20.16	$\mathbf {< 0.001}$	0.43
20% Noise	1, 27	4.04	0.055	0.13	1, 27	16.49	$\mathbf {< 0.001}$	0.38
40% Noise	1, 27	15.51	$\mathbf {< 0.001}$	0.36	1, 27	0.08	0.774	0.003
Noise level	2.23, 60.12$^\dagger$	73.69	$\mathbf {< 0.001^\dagger }$	0.73	2.27, 61.29$^\dagger$	3.55	$\mathbf {0.030^\dagger }$	0.12
Modality x Noise level	2.80, 75.59$^\dagger$	2.43	0.076$^\dagger$	0.08	4, 108	9.46	$\mathbf {< 0.001~}$	0.26

*Note*: *df* = degrees of freedom. $\eta ^2_p$ = partial $\eta ^{2}$. Bold *p* values indicate statistical significance ($p < 0.05$).

$^\dagger$Greenhouse-Geisser-corrected *df* and *p*.

Subjects’ performance was defined by non-parametric estimates of sensitivity (*A*) and decision criterion (*b*), computed using the method proposed by Zhang and Mueller (2005). The choice of non-parametric rather than parametric measures was dictated by the fact that for all noise levels except 0%, IPIs for 4- and 6-Hz stimuli came from distributions of unequal variance (see Fig. 2). We calculated subject-level *A* and *b* estimates separately for each of the 10 conditions (2 modalities$~\times ~$5 noise levels). For these estimates, a hit was defined as a response of “slow” to a 4-Hz stimulus, and a false alarm as a response of “slow” to a 6-Hz stimulus. A higher value of *A* indicates greater accuracy in categorizing stimulus rate. The value of *b* reflects the location of a subject’s criterion for deciding whether a stimulus rate was 4 or 6 Hz, and therefore the relative probability of each possible misattribution error. Specifically, a value of $b=1$ means that the subject used a neutral criterion, favoring neither response; that is, $b=1$ means that both types of misattributions were equally likely. $b<1$ signifies that mistaken “slow” responses exceeded mistaken "fast" responses, and $b>1$ signifies that mistaken “fast” responses exceeded mistaken "slow" responses.

Separate within-subject, two-way omnibus ANOVAs (*afex* package; Singmann et al. , 2022) tested for differences between modalities and among noise levels for both *A* and *b*. We evaluated sphericity across noise levels using Mauchly’s test; violations were corrected with Greenhouse-Geisser $\mathcal {E}$ (Verma, 2015). Using the *emmeans* package (Lenth, 2022), we tested for a simple effect of modality in each noise level, for both dependent variables. Additionally, we tested whether $b=1$ in each condition using single-sample *t*-tests, adjusting *p*-values and confidence intervals with a Bonferroni correction for 10 estimates.

**Table 2:** Decision criterion (*b*) by condition, compared to the neutral value, $b=1$.

	Visual			Tactile		
Noise level	M (95% CI)$^\dagger$	*p*$^\dagger$	$\eta ^2_p$	M (95% CI)$^\dagger$	*p*$^\dagger$	$\eta ^2_p$
0%	1.42 (1.07, 1.77)	$\mathbf {0.012}$	0.33	1.15 (0.94, 1.37)	0.353	0.15
5%	1.45 (1.11, 1.80)	$\mathbf {0.004}$	0.37	1.09 (0.87, 1.31)	1.000	0.06
10%	1.51 (1.21, 1.82)	$\mathbf {< 0.001}$	0.49	1.08 (0.84, 1.33)	1.000	0.04
20%	1.54 (1.22, 1.85)	$\mathbf {< 0.001}$	0.50	1.13 (0.88, 1.39)	1.000	0.09
40%	1.12 (0.88, 1.35)	1.000	0.08	1.14 (0.88, 1.40)	1.000	0.10

*Note*: M: estimated marginal mean; CI: confidence interval. Null hypothesis: $b = 1$.

$^\dagger$Confidence intervals and *p*-values adjusted using Bonferroni method for 10 estimates.

**Fig. 4:**
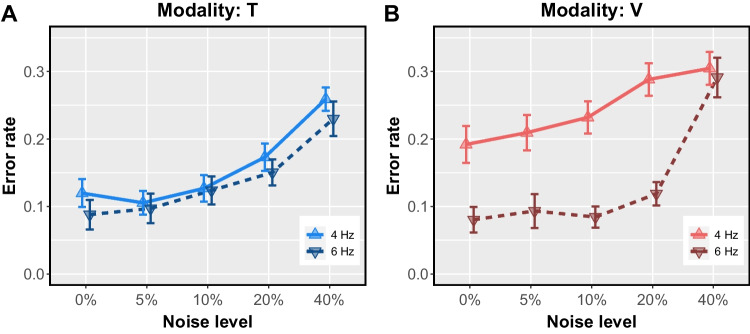
Error rate for each combination of modality and noise level. **A**. Subjects had similar accuracy on 4-Hz and 6-Hz **T** trials. **B**. Subjects made more errors on 4-Hz **V** trials than 6-Hz **V** trials, that is, they were more likely to label a **V** pulse rate as “fast” overall, regardless of nominal rate. Error bars reflect within-subject standard error, n=28.

### Results

Figure 3A shows mean sensitivity (*A*) for each combination of noise level and modality. As expected, on average, sensitivity significantly declined as noise increased (Table 1). Additionally, modality had a significant effect on sensitivity (Table 1), with higher **T** sensitivity compared to **V**, on average (Fig. 3A). *Post hoc *tests for a simple effect of modality at each noise level showed significantly better sensitivity on **T** trials than on **V** trials with 5% noise (mean difference = 0.029, 95% CI: [0.010, 0.047]), and 40% noise (mean diff. = 0.05, 95% CI: [0.024, 0.076]). Sensitivity did not significantly differ between modalities with 0%, 10%, or 20% noise (Table 1). Despite significant modality effects at some but not all of the noise levels, we did not observe a significant interaction between modality and noise level (Table 1).

Figure 3B shows the mean decision criterion (*b*) in each condition. A within-subjects ANOVA yielded a significant main effect of modality (Table 1). Subjects used significantly different decision criteria between modalities on trials with low noise, that is, 0-20% noise (Table 1). Over that range, the mean difference in criterion ($b_{diff} = b_T - b_V$) was, at each noise level: 0% noise $b_{diff}$ = -0.265, 95% CI: (-0.434, -0.096); 5% noise = -0.364 (-0.560, -0.168); 10% noise = -0.427 (-0.623, -0.232); 20% noise = -0.403 (-0.607, -0.200) (see Table 1 for *p*-values and effect sizes). On these low-noise trials, subjects’ mean **V** criterion significantly differed from neutral ($b_V \ne$ 1, Table 2). With the highest noise level (40%), subjects’ mean **V** criterion shifted toward neutral, *b* = 1 (Table 2), similar to subjects’ mean **T** criterion ($b_{diff}$ = 0.029, 95% CI [-0.176, 0.234]; Table 1, Fig. 3B). Subjects used a fairly consistent decision criterion on **T** trials over all five noise levels (blue line in Fig. 3B). Although that criterion tended to favor "fast" responses (that is, $b_T>$ 1), it did not significantly differ from neutral (*b* = 1) in any of the five noise levels (Table 2).

### Experiment 1 Discussion

#### Sensitivity

Adding random temporal noise to pulse trains impaired categorization of rate, and did so similarly for both **V** and **T** stimuli. On average, however, vibrotactile rate was more accurately categorized than visual rate, regardless of how much variability was injected into the signal. Previous comparisons of visual and tactile temporal pattern perception produced comparable results, that is, temporal patterns presented as tactile stimuli are processed more accurately than patterns presented as visual stimuli (Espinoza-Monroy & de Lafuente, 2021; Kang et al., 2018). For example, Kang et al., (2018) found that subjects could detect repeats of random pulsatile patterns better with tactile pulses than with visual pulses. The authors also demonstrated that perceptual learning of temporal patterns was more likely to occur with tactile than with visual pulse sequences (Kang et al., 2018). Our study extends these findings to the case of rate categorization, and demonstrates that with considerable temporal variability, subjects categorize vibrotactile rate more accurately than visual rate.

#### Decision Criterion

The difference in decision criterion between modalities may be best understood in terms of tendency to make errors in judging each rate category, 4 Hz and 6 Hz. Specifically, when *b* = 1, the neutral criterion value, errors were equally likely for both stimuli; when $b>1$, 4-Hz stimuli were more likely to be deemed 6-Hz than the reverse. Figure 4 depicts error rates by modality. Subjects made similar proportions of errors on 4-Hz **T** trials and 6-Hz **T** trials (Fig. 4A), suggesting that a neutral, unbiased decision criterion was used on **T** trials regardless of noise level ($b_T \sim 1$, Table 2, Fig. 3B). Subjects used a different criterion on most **V** trials, one that reflects the higher error rate on 4-Hz **V** trials compared to 6-Hz **V** trials (Fig. 4B). In other words, on **V** trials, subjects were more likely to incorrectly label a 4-Hz **V** stimulus "fast" than they were to incorrectly label a 6-Hz **V** stimulus "slow".

One potential interpretation of this finding is that most **V** pulse sequences tended to appear “fast” to subjects, regardless of the nominal rate. Studies of perceived interval duration across sensory modalities provide indirect support for this possibility. For example, with physical duration equated, visual stimuli tend to appear to last longer than than tactile stimuli (Azari et al., 2020; Jones et al., 2009; Tomassini et al., 2011). If the perceptual response to an individual **V** pulse lasted longer than the perceptual response to a **T** pulse, then the unfilled IPIs between **V** pulses may have appeared *shorter* than their **T** counterparts, increasing the likelihood that both 4- and 6-Hz **V** stimuli would tend to seem fast. While the complexity of this experiment’s stimuli and task foreclosed testing the proposition that individual **V** pulses seem to last longer than individual **T** pulses, we expand upon the potential role of sensory persistence in the General Discussion.

## Experiment 2: Unimodal and Bimodal Temporal Sensitivity

For another perspective on temporal resolution across visual and tactile pulse perception, we measured thresholds for detecting an unfilled interval between two successive visual pulses and two successive vibrotactile pulses. Guided by the results of Experiment 1, where temporal acuity was poorer with visual stimuli, we expected higher **V** gap detection thresholds in Experiment 2.

In addition to measuring gap detection thresholds for unimodal **V** and **T** pulses, we measured the gap detection threshold for bimodal stimuli comprising temporally coterminous **T** and **V** pulses. The pooling or combination of information from multiple sources has long been among neuroscience’s major concerns (Chandrasekaran, 2017; Parker, 1998; Sinnett et al., 2008). When those distinct sources are multiple sensory modalities, their interaction can take many different forms, including facilitative combination (Forster et al., 2002; Sperdin et al., 2009), destructive competition (Sinnett et al., 2008; Sun & Sekuler, 2021), compromise (Crommett et al., 2019), and unimodal dominance (Burr et al., 2009; Colavita, 1974; Villalonga et al., 2021). With one exception, these studies did not explicitly require temporal judgments. The exception, Villalonga et al., (2021), measured subjects’ ability to categorize the temporal deviation of a single pulse presented after an isochronous series of pulses. Pulses were either auditory, vibrotactile, or a bimodal combination of the two. Temporal acuity for the auditory stimulus was superior to acuity for the timing of the vibrotactile pulse. Importantly, performance with bimodal stimuli seemed to be dominated by the more reliably-categorized auditory stimulus. Although the stimulus structure and task demands in Villalonga et al. ’s study distinguished it from the paradigm we used in Experiment 2, which entailed just two pulses per trial, both tasks have some dependence on temporal acuity. That connection led us to predict that in our task, too, the stimulus from the modality with better temporal acuity would dominate bimodal performance.

### Method

#### Subjects

Fourteen subjects (11 female, 2 male, 1 declined to identify; mean age = 22.3 years, SD = 3.4) participated in the experiment. With n=14 subjects, we had 99.9% power to observe an effect size reported in a similar visuo-tactile task ($\eta _p^2 = 0.45$; Bultitude et al. , 2016). Experimental procedures were approved by Brandeis University’s Institutional Review Board and were conducted in accordance with the Declaration of Helsinki. Subjects gave written informed consent prior to participation, and none had participated in Experiment 1.

#### Apparatus and Stimuli

Using the same apparatus as in Experiment 1 (Fig. 1), MATLAB and the Psychophysics Toolbox controlled stimulus presentation and collected responses (Brainard, 1997; Kleiner et al., 2007; Matlab, 2017; Pelli, 1997). In addition to the same **V** and **T** stimuli as in Experiment 1, we included a third condition in which concurrent **V** and **T** components were paired to produce a bimodal, visuo-tactile (**VT**) stimulus. In the **VT** condition, coterminous unimodal components were presented together.

**Fig. 5:**
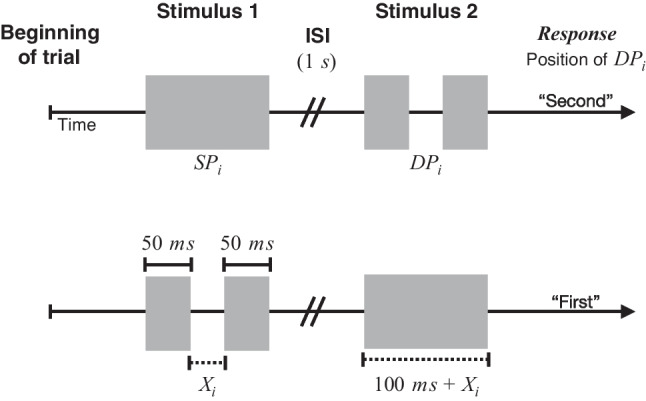
Diagram of the trial structure in Experiment 2. Subjects received either a stimulus comprising a single, continuous pulse ($\varvec{SP_i}$) followed by a double-pulse stimulus ($\varvec{DP_i}$; top), or $\varvec{DP_i}$ followed by $\varvec{SP_i}$ (bottom). The IPI $\varvec{X_i}$ separating pulses in $\varvec{DP_i}$ ranged 2-32 ms. All pulses presented in a single trial came from the same modality condition (**V**, **T**, or **VT**). After the second stimulus, subjects had up to 2 s to indicate whether $\varvec{DP_i}$ had occurred first or second

#### Task

A 2IFC discrimination paradigm determined the temporal threshold for detecting a brief, unfilled interval between successive stimulus pulses (Fig. 5). A 200-ms long tone announced the beginning of a trial. Then, 800 ms later, two different stimuli were presented in random order. One was the “double-pulse” stimulus ($\varvec{DP_i}$), comprising two 50-ms pulses separated by an empty IPI with duration $= \varvec{X_i}$; the other was the “single-pulse” stimulus ($\varvec{SP_i}$) comprising a single uninterrupted pulse whose total duration matched the total duration of the accompanying double pulse stimulus. Note that, to ensure that differences in total stimulus duration was not correlated with $\varvec{X_i}$, the duration of $\varvec{SP_i}$ was always equal to the total duration of $\varvec{DP_i}$ = 100 ms + $\varvec{X_i}$. For each trial *i*, the value of $\varvec{X_i}$ was drawn randomly from a discrete uniform distribution of five different gap durations, $\varvec{X_i}= \{ 2, 4, 8, 16, 32 \}$ ms. Previous research (Desloge et al., 2014; Gescheider et al., 2003; Van Doren et al., 1990) and our own preliminary testing suggested that this range would likely encompass the thresholds across conditions. Both stimuli, $\varvec{DP_i}$ and $\varvec{SP_i}$, were always presented in the same modality on each trial (**V** , **T** , or **VT**), and were separated by an inter-stimulus interval of 1 s. The subject indicated whether $\varvec{DP_i}$ occurred first or second on that trial, using the computer keyboard, as in Experiment 1, with response key mappings counterbalanced across subjects. Following the termination of the second stimulus, subjects had up to 2 s to respond (shortened from Experiment 1 in which subjects responded on average in well under 2 s). Trials were separated by an inter-trial interval of 0.5 s.

Subjects completed one practice block of 10 trials per modality condition, with the option of repeating each practice block until they felt confident to proceed with the task. Trials in the practice block were graded in difficulty, beginning with four trials where $\varvec{X_i} = 50$ ms and ending with six trials where $\varvec{X_i} = 32$ ms (the easiest experimental condition). Test trials, block randomized by modality (**V** , **T** , or **VT**), were presented in blocks of 50 trials with stimulus order and gap duration randomized from trial to trial. During the break between blocks, subjects received feedback in the form of percent correct for the last completed block, and were informed which modality would be tested next. Subjects completed a total of three test blocks in each modality, producing a total of 450 test trials per subject (30 trials per gap duration, per modality condition).

#### Data Analysis.

Data were analyzed in R (R Core Team, 2021). As in Experiment 1, we excluded data from trials where subjects failed to respond (57 trials, 0.9% of all trials). For two reasons, we did not exclude any trials based on premature responding. First, our 2IFC design may have led to very short RTs on trials in which $\varvec{DP_i}$ appeared first. Second, when we examined the distribution of responses on trials with RT < 100 ms (96 trials, 1.5% of the data), the majority of these responses occurred on trials with the easiest (*i.e.*, supra-threshold) conditions. Reasoning that these trials produced valid measurements, we chose not to exclude them from analysis. Following exclusions, the remaining data were aggregated by subject and condition.

To estimate gap detection threshold in each modality condition, we fit group-level, modality-specific logistic psychometric functions (PFs) using the *glm* function in R, with a 2AFC binomial link function from R’s *psyphy* package (Knoblauch, 2002; Knoblauch & Maloney, 2012). We also fit subject-level PFs for each condition, using the *glmer* function from R’s *lme4* package (Bates et al., 2015). In the subject-level model, we included intercepts and slopes as random effects. We estimated each modality’s gap detection threshold by taking the inverse of the group-level PF at $p = 0.76$. This defined the gap duration that would produce 76% correct ($d' = 1$; Klein , 2001). To generate the standard error for each threshold, we generated three bootstrap distributions (one for each modality-specific threshold) with 5,000 re-samples, using the *boot* function from R’s *boot* package (Cantry & Ripley, 2021). To compare unimodal to bimodal performance and test our hypothesis that the more sensitive modality would dominate bimodal judgments, we used Pearson’s product-moment correlation tests to compare individual subjects’ mean accuracy from each unimodal condition to the bimodal condition.

### Results

**Fig. 6:**
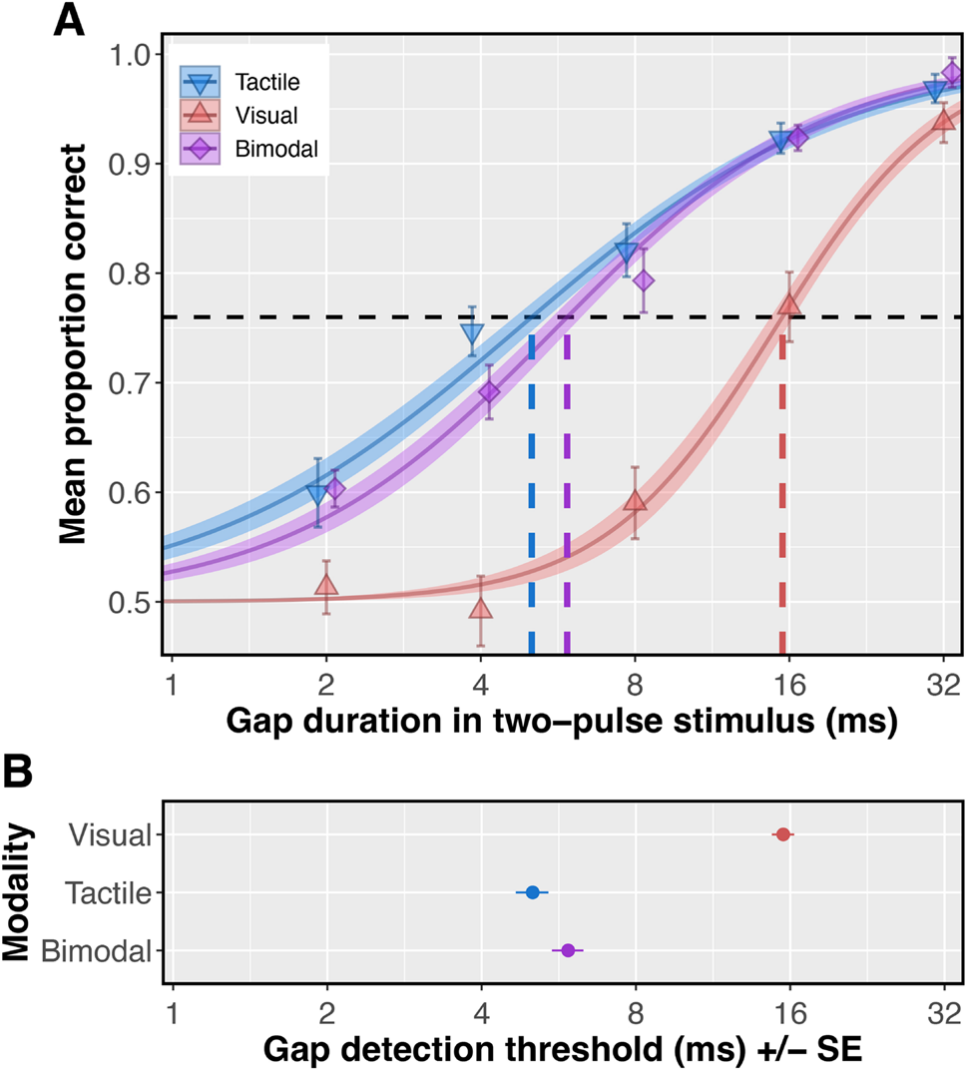
Psychometric modeling of Experiment 2 data. **A**: Group-level logistic psychometric functions (PFs) fit using maximum likelihood estimation. Ribbons around each PF represent the PF’s standard error. Points show the mean measured accuracy in each condition; error bars around each point represent within-subject standard error. Dashed vertical lines denote the estimated sensory threshold in each modality, which we defined at 76% correct ($d' = 1$; black dashed horizontal line). **B:** Average thresholds and bootstrapped standard errors for **V**, **T**, and **VT** stimuli.

Psychometric modeling revealed differences in the two-pulse thresholds for **V**, **T**, and **VT** stimuli. Group-level gap detection thresholds for the three types of stimuli are shown by the dashed vertical lines in Fig. 6A, and again, with error bars added, by the points in Fig. 6B. Subject-level PFs followed the same overall trend as the group model (see Supplementary Fig. 1). The estimated **V** gap threshold was 15.5 ms (SE: 0.78 ms). In contrast, the estimated **T** gap threshold was 5.0 ms (SE: 0.37 ms), and the estimated **VT** threshold was 5.9 ms (SE: 0.42 ms).

**Fig. 7:**
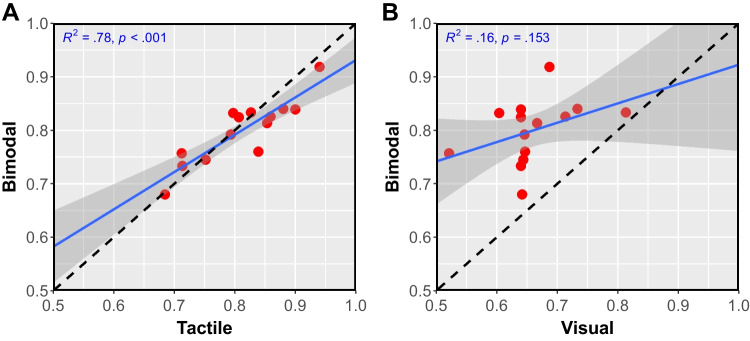
Correlation of subject-level unimodal and bimodal performance in Experiment 2. **A**: Tactile vs. bimodal accuracy. **B**: Visual vs. bimodal accuracy. Points are mean accuracy for individual subjects. The dashed black line represents the value expected if bimodal performance (on the vertical axis) were determined entirely by the unimodal performance (on the horizontal axis). Also shown in each panel is the 95% confidence region around the maximum likelihood line of best fit.

To further examine performance at the level of individual subjects, we calculated each subject’s mean accuracy over all five levels of $\varvec{X_i}$, for each modality condition separately. Subjects were most accurate on **T** trials (mean accuracy 81.12%, 0.99% within-subject standard error [WSSE]), followed by **VT** trials (79.95%, 0.91% WSSE), and least accurate on **V** trials (65.98%, 1.49% WSSE). Subject-level **T** accuracy was strongly correlated with **VT** accuracy, $R^2$ = 0.78, *p* < 0.001. In contrast, subject-level **V** accuracy was not significantly correlated with **VT** accuracy, $R^2$ = 0.16, *p* = 0.153. Figure 7 shows the results for these correlations. To test whether bimodal performance might be attributable to complete dominance by either unimodal cue, we compared the slope of the best-fit maximum likelihood line (shown in blue in Fig. 7) to 1.0, the theoretical case for unimodal cue dominance (shown in black in Fig. 7). Both slopes significantly differed from 1 (**T**: $\beta$ = 0.698, *p* = 0.015; **V**: $\beta$ = 0.429, *p* = 0.020). Taken together, these results suggest that even though there was not complete dominance by either unimodal cue in the bimodal percept, **T** performance was the stronger predictor of **VT** performance.

### Experiment 2 Discussion

We found subjects’ **V** thresholds to be nearly 3$\times$ their **T** thresholds (Fig. 6), showing that the **T** modality supports finer temporal acuity. For example, as Fig. 6A shows, subjects would be able detect a 5-ms unfilled interval separating two **T** pulses more than 75% of the time, but would not perform any better than the guess rate with **V** pulses separated by a gap of the same length. Subjects’ mean accuracy in each modality condition also reflected this difference, with highest accuracy on unimodal **T** trials and lowest accuracy on unimodal **V** trials.

As mentioned above, performance in **T** and **VT** conditions was much more strongly correlated than was performance in **V** and **VT** conditions. This difference strengthens the conclusion we drew from Fig. 6A, namely that with bimodal stimuli, subjects based their judgments largely on the tactile component of the stimulus. This finding can be interpreted as an analog of what Barlow called “the lower envelope principle” (Barlow, 1972, 1995). That principle states that the behavioral threshold is determined entirely by the most sensitive of multiple sensory responses: “Sensory thresholds are set by the class of sensory unit that has the lowest threshold for the particular stimulus used and are little influenced by the presence or absence of responses in the enormous number of other neurons that are less sensitive to the stimulus” (Barlow, 1995, p. 418). In our experiment, the **VT** threshold appears to have been set *predominantly, but not entirely,* by **T** inputs: that the slope of the predicted **T** line differs from 1 (Fig. 7A) shows there was not complete **T** dominance.

## General Discussion

A central question drove this study: How does temporal information processing differ between tactile and visual sensory modalities? Using two convergent approaches, rate categorization and gap detection, we demonstrated that human subjects are more sensitive to temporal information conveyed by brief pulses of vibrotactile stimulation, compared to visual flashes. Experiment 1 provided clear evidence of modality-related differences in rate categorization; Experiment 2 demonstrated a possible cause of that result, namely that subjects perceive unfilled intervals marked by individual tactile pulses with better temporal accuracy than they do with visual pulses. In the following section, we compare our findings to those of other studies of sensory timing and consider the implications of our results.

### Interval and Pattern Timing

That temporal sensitivity for tactile stimulation is better than temporal sensitivity to visual stimulation is not a novel idea. Empirical support for that idea comes largely from measurements of perceived duration of single, isolated intervals (Azari et al., 2020; Ball et al., 2017; Grondin, 2014; Tomassini et al., 2011; van Erp Jan & Werkhoven, 2004), and results from our Experiment 2 add to this body of support. Our study additionally demonstrates that this difference between modalities is not limited to the single interval duration judgments that so many previous studies focused on. With Experiment 1, we extend that conclusion to judgments of the average rate of a *sequence* of intervals. The distinction between judgments of single versus sequences of intervals has particular importance in neural models of sub-second timing that distinguish between timing of simple intervals and timing of more complex temporal patterns (Hardy & Buonomano, 2016). Different task demands can recruit different distributed timing mechanisms (Bouwer et al., 2020; Buhusi & Meck, 2005; Levitan et al., 2015; Merchant et al., 2008; Paton & Buonomano, 2018). While we found consistent tactile superiority in pattern timing (Experiment 1) and interval timing (Experiment 2) tasks, we cannot determine from the present study whether the modality difference in both tasks arose from shared or distinct underlying timing mechanisms, or some combination thereof.

Despite this limitation, one possible influence shared between both experiments is sensory persistence. It is well known that the response to a brief visual stimulus outlasts the stimulus itself (*e.g.*, Kelly , 1971; Sperling , 1960), a persistence that is reflected in vision’s temporal impulse response function (Georgeson, 1987; Ikeda, 1986; Nilsson, 1979). Because of persistence, two pulses separated by a sufficiently short empty interval may be perceived as a single, continuous, uninterrupted pulse. Neural persistence has also been documented in the tactile modality, specifically in the Pacinian channel targeted by 250-Hz vibrations (Gescheider et al., 1999, 2003), but how the time course of tactile persistence compares to that of visual persistence is unknown. The shorter tactile gap detection threshold we observed in Experiment 2 supports the idea that tactile persistence was briefer than its visual counterpart. A longer visual persistence could also explain the difference in error types that was specific to visual rate judgments in Experiment 1. If IPIs appeared truncated on **V** trials compared to **T** trials, subjects would have made more ”fast“ judgments on **V** trials overall, leading to higher error rates on 4-Hz than 6-Hz **V** trials, as we observed (Fig. 4).

The decision to employ a binary categorization of rate in Experiment 1 limited the inferences about temporal processing that the experiment could support. Even with sequences that were corrupted by considerable noise, subjects succeeded in reliably distinguishing between two rates. Obviously, the two rates included in our task do not tile the space of all possible temporal patterns that can be applied to the skin. As a result, the experiment leaves wide open the possibility that many other types of temporal patterns can be successfully categorized and used to communicate information about the environment, when either applied to the skin or presented visually. For example, one simple extension of Experiment 1 might entail discrimination of frequency-modulated signals, such as pulse trains that systematically increase or decrease in frequency (*i.e.*, frequency sweeps; Crommett et al. , 2019; Yi & Sekule, 2022). Also deserving of investigation is whether tactile perception would outperform visual perception with more complex temporal patterns, like periodic rhythmic structures (Bouwer et al., 2020) or random patterns of stimulation (Kang et al., 2018). Importantly, to test the limits of tactile stimulation to transmit information via temporal patterns, future research might also determine the degree to which vibrotactile signal intensity, which we held constant, interacts with temporal perception (Sharma et al., 2022), as well as with mechanisms of perceptual learning and memory for temporal sequences (Gold et al., 2014; Kang et al., 2018).

### Cross-Modal Timing Interactions

Results from these experiments have multiple implications for multisensory integration research. Tactile signals were perceived with better temporal resolution than visual signals in both experiments, and in Experiment 2 subjects largely based their judgments about bimodal stimuli upon the tactile component of the stimulus. Consistent with the modality appropriateness hypothesis (Welch & Warren, 1980), these findings suggest that vibrotactile perception is better suited for processing temporal information than the visual system, which has importance in the framework of sensory dominance. It is well documented, for example, that auditory signals influence visual perception in the temporal domain due to audition’s superior temporal acuity (Burr et al., 2009; Recanzone, 2003; Shimojo & Shams, 2001; Shipley, 1964). Similarly, tactile temporal processing may drive temporal perception of visuotactile stimuli, given its demonstrated unimodal advantage.

Our results support previous studies that have reported touch-induced bias on visual perception (Bresciani et al., 2008; Violentyev et al., 2005). In these studies, subjects were instructed to attend to and report the number of visual flashes they perceived, while ignoring a concurrent sequence of tactile pulses. A notable difference between these and the current study is that subjects were not instructed to attend to either unimodal component while ignoring the other: subjects were instructed to use both channels of sensory information to make their responses. When studying cross-modal influences on counting judgments (Werkhoven et al., 2009), the influence of vision on touch was stronger than the influence of touch on vision when subjects were not instructed ahead of time to attend to a particular modality. While our Experiment 2 results contradict this finding, we were limited by our decision to always use congruent bimodal pairings (*i.e.*, the number of tactile pulses always matched the number of visual pulses). It will be important for future work to include both congruent and incongruent bimodal conditions, as well as to explore the effects of attention on visuo-tactile integration of temporal information.

### Locus and Type of Tactile Stimulation

In Experiments 1 and 2, vibrotactile stimuli were delivered only to the distal tip of the left index finger. We chose that site because of its particular sensitivity to light touch (Sekuler et al., 1973; Weinstein & Kenshalo, 1968). Many other studies of tactile perception have also used the index finger as the locus of stimulation (Bresciani et al., 2008; Crommett et al., 2019; Espinoza-Monroy & de Lafuente, 2021; Fujisaki & Nishida, 2009; Machulla & Ernst, 2016), with some others extending stimulation to other fingertips (Ball et al., 2017; Kang et al., 2018; Rahman et al., 2020) or the entire hand (Azari et al., 2020; Tomassini et al., 2011). Additionally, these investigations of tactile temporal sensitivity have utilized not just vibrotactile stimulation, but also electrocutaneous stimulation and individual taps. Variations in the type of tactile stimulation used across studies may have led to the mixed results of previous visuo-tactile comparisons. A unique aspect of tactile stimulation is that different forms of tactile information could be delivered simultaneously to multiple distributed loci. Comparable knowledge about various body parts’ sensitivity to stimulus timing is lacking; the absence of such data means we must be cautious about extrapolating from our results to other loci of stimulation and forms of tactile stimulation (Dim & Ren, 2017).

One potential application of the present research is in the development of devices that use vibrotactile stimulation to convey important information to users (Choi & Kuchenbecker, 2013; Janidarmian et al., 2022). Investigating sensitivity to tactile temporal patterns of stimulation at various loci could greatly expand the communicative potential of vibrotactile signaling, beyond a basic alerting function (Chiasson et al., 2003). Future investigations should proceed with careful attention to the density of Pacinian corpuscles at each potential locus of stimulation, which could create variation in temporal acuity to pulsatile vibrations (Choi & Kuchenbecker, 2013), as well as effects of aging, which has been shown to impact neural persistence (Gescheider et al., 2003; Humes et al., 2009; Van Doren et al., 1990) and mechanisms of multisensory integration (de Dieuleveult et al., 2017; Poliakoff et al., 2006).

### Conclusion

Our study adds to a growing literature on the modality-dependent nature of different forms of temporal processing. Studies of this kind gain importance from the widespread implementation of multimodal pulse rates and other temporal patterns in communication devices for individuals with sensory deficits (Choi & Kuchenbecker, 2013). As designers of these devices look to incorporate vibrotactile signals into existing displays, gauging the information-carrying potential of this understudied modality is crucial. Understanding how temporal information is processed when delivered via different sensory modalities is not only important from a device-engineering perspective: it also gives us valuable insight into how the brain perceives time (Bratzke & Ulrich, 2019; Buonomano & Karmarkar, 2002; Ivry & Spencer, 2004). Work in this domain has clinical importance, for time-keeping abilities are impaired in some neurological disorders, such as schizophrenia and Parkinson’s disease (Gu et al., 2015; Penney et al., 2005; Tracy et al., 1998). For these reasons, ongoing research on time perception seeks to establish a “taxonomy of perceptual time” (Meck & Ivry, 2016; Paton & Buonomano, 2018), a classification scheme that defines and differentiates various forms of perceptual timing based on underlying neural mechanisms. Overall, then, our findings point to the value of further study of visuo-tactile timing, and multisensory timing more broadly.

### Supplementary Information

Below is the link to the electronic supplementary material.

Supplementary file 1 (pdf 599 KB)

## Data Availability

The data analyzed in the current study as well as the scripts used for statistical analysis can be found at: https://osf.io/u2jt3/?view_only=a39c3428d2fd445b80897d8915b2857f
