# Supplementary material for: Keep your finger on the pulse: Better rate perception and gap detection with vibrotactile compared to visual stimuli
Source: Atten Percept Psychophys. 2023 Aug 16;85(6):2004–17. doi: 10.3758/s13414-023-02736-y (PMC10545646; doi:10.3758/s13414-023-02736-y)
Supplement: Supplementary file 1 — (pdf 599 KB) [file 13414_2023_2736_MOESM1_ESM.pdf]

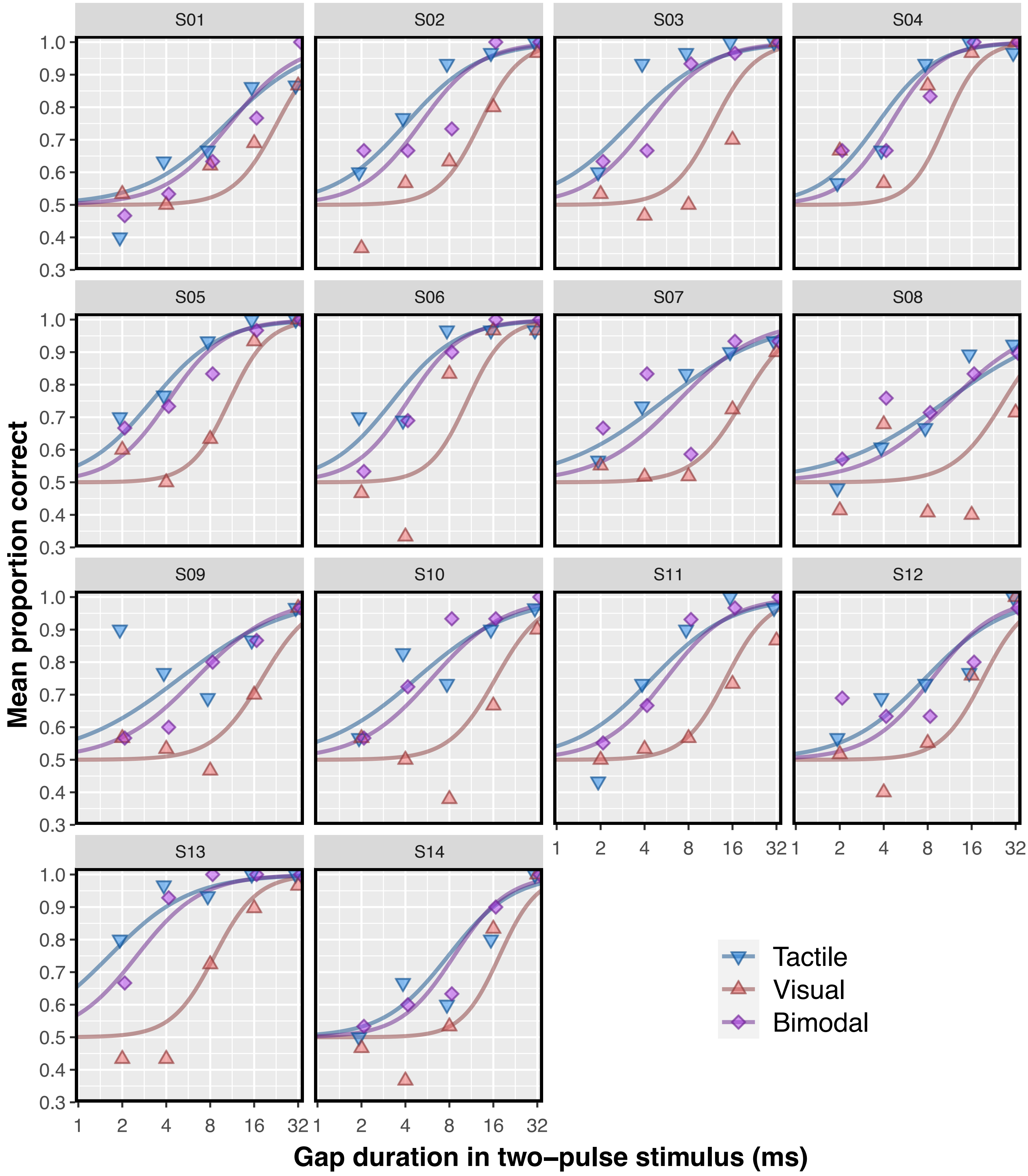


**Supplementary Figure 1**. Psychometric functions (PFs) for each individual subject (n=14) in Experiment 2. We fit PFs to psychophysical data using a multivariate generalized linear mixed model, including intercepts and slopes as random effects. For all subjects, a clear rightward shift of the visual PF (red) is evident, while tactile (blue) and bimodal (purple) PFs largely overlap, consistent with the group-level PF shown in Fig. 6 of the main text.
